# Head inversion technique to restore physiological conjunctival structure for surgical treatment of primary pterygium

**DOI:** 10.1038/s41598-018-35121-z

**Published:** 2018-11-09

**Authors:** Fumiaki Yoshitomi, Tetsuro Oshika

**Affiliations:** 1 Yoshitomi Eye Center, Fukuoka, Japan; 2 Department of Ophthalmology, Faculty of Medicine, University of Tsukuba, Ibaraki, Japan

## Abstract

We describe a new surgical technique to treat primary pterygium, the head inversion technique, with its surgical outcomes. Seventy-five eyes of 75 consecutive patients with primary pterygium undergoing surgical treatment were included. The pterygium head and body were bluntly separated from the cornea and inverted onto the nasal conjunctival area. By injecting a balanced salt solution subconjunctivally, the conjunctiva was ballooned and smoothed. Two or three interrupted 8–0 virgin silk sutures were placed to secure the inverted conjunctiva in place. No adjunctive therapy was used during and after surgery. Postoperatively, one eye showed pterygium recurrence at 233 days, in which an unintended conjunctival hole was made during surgery. The Kaplan-Meier analysis showed that the recurrence rate at 1 year was 2.4%. In 43 eyes which were followed up for longer than 6 months, the vascular loop, which is characteristic of normal limbal structure, appeared on the nasal conjunctiva in 41 eyes (95.3%). The palisades of Vogt were found on the nasal limbus postoperatively in 13 eyes (30.2%). The pterygium head inversion technique was an effective treatment for primary pterygium. By separating the pterygium from the cornea and inverting the intact pterygium head onto the nasal conjunctival site, the conjunctiva restored near physiological status after surgery.

## Introduction

A pterygium is a triangular, elevated, superficial, fibrovascular lesion that usually forms over the perilimbal conjunctiva and encroaches onto the corneal surface. Exposure to sunlight, older age, male gender, outdoors occupation, and living in rural environments are the leading risk factors for the development of pterygium^1^. Pterygia continue to be a source of local irritation, cosmetic aggravation, and visual disturbance from a patient perspective. They also remain ophthalmic enigmas for physicians due to lack of exact understanding of the actual pathogenic mechanisms and propensity to recur after surgical excision^2,3^.

The fact that various surgical approaches exist for the treatment of pterygium underscores the reality that no single technique is universally satisfactory in terms of recurrence rate, complication, technical simplicity, and cosmetic outcomes. The simplest surgical method for pterygium treatment is bare sclera excision, leaving the bulbar conjunctival defect after pterygium removal uncovered and letting the surrounding conjunctiva migrate over the area of exposed sclera on its own. This technique was, however, associated with high recurrence rate^4,5^, and thus replaced by other more complicated and sophisticated methods. Such surgical strategies were developed based on the premise that close approximation of healthy conjunctival tissue at the denuded limbus after pterygium removal will prevents recurrences. The three basic variations on this theme include excision with primary conjunctival closure, excision with conjunctival flap formation, and conjunctival autograft.

These procedures, however, still cannot restore the natural, healthy conjunctival structures. Surgical excision, closure or grafting, and adjunctive therapy will inevitably affect the physiological status of the conjunctiva and compromise conjunctival barrier, which will not be fully recovered. To overcome these drawbacks, we developed a new technique for the treatment of pterygium. Our method does not entail excision and incision of pterygium, but attempts to restore conjunctival structure and barrier function to the normal condition as much as possible.

## Patients and Methods

### Patients

Seventy-five eyes of 75 consecutive patients with primary pterygium undergoing surgical treatment between November 2014 to September 2015 were recruited. Eyes with recurrent pterygium were not included. When bilateral eyes of one patient were operated on, only the first eye was included in the analyses. There were 30 males and 45 females, and their age averaged 73.0 ± 7.6 years old (rage, 57–89 years old). The degree of pterygium was graded according to the preoperative size (location of the pterygium head relative to the corneal radius)^6^. The pterygium head was located within 1/3 of the corneal radius from the limbus in 17 eyes (22.7%), the head reached between 1/3 and 2/3 of the corneal radius in 52 eyes (69.3%), and the pterygium end position exceeded 2/3 of the corneal radius from the limbus toward the corneal center in 6 eyes (8.0%). Among 75 patients, there were 9 cases with pollinosis, 4 cases with diabetes mellitus, 2 cases with dry eye, 2 cases with collagen disease (systemic lupus erythematosus and autoimmune pancreatitis), 1 case with osteoporosis, and 1 case with gout. After pterygium surgery, the patients were followed up for an average of 273 ± 193 days (range, 7–688 days).

### Surgery

**Figure 1:**
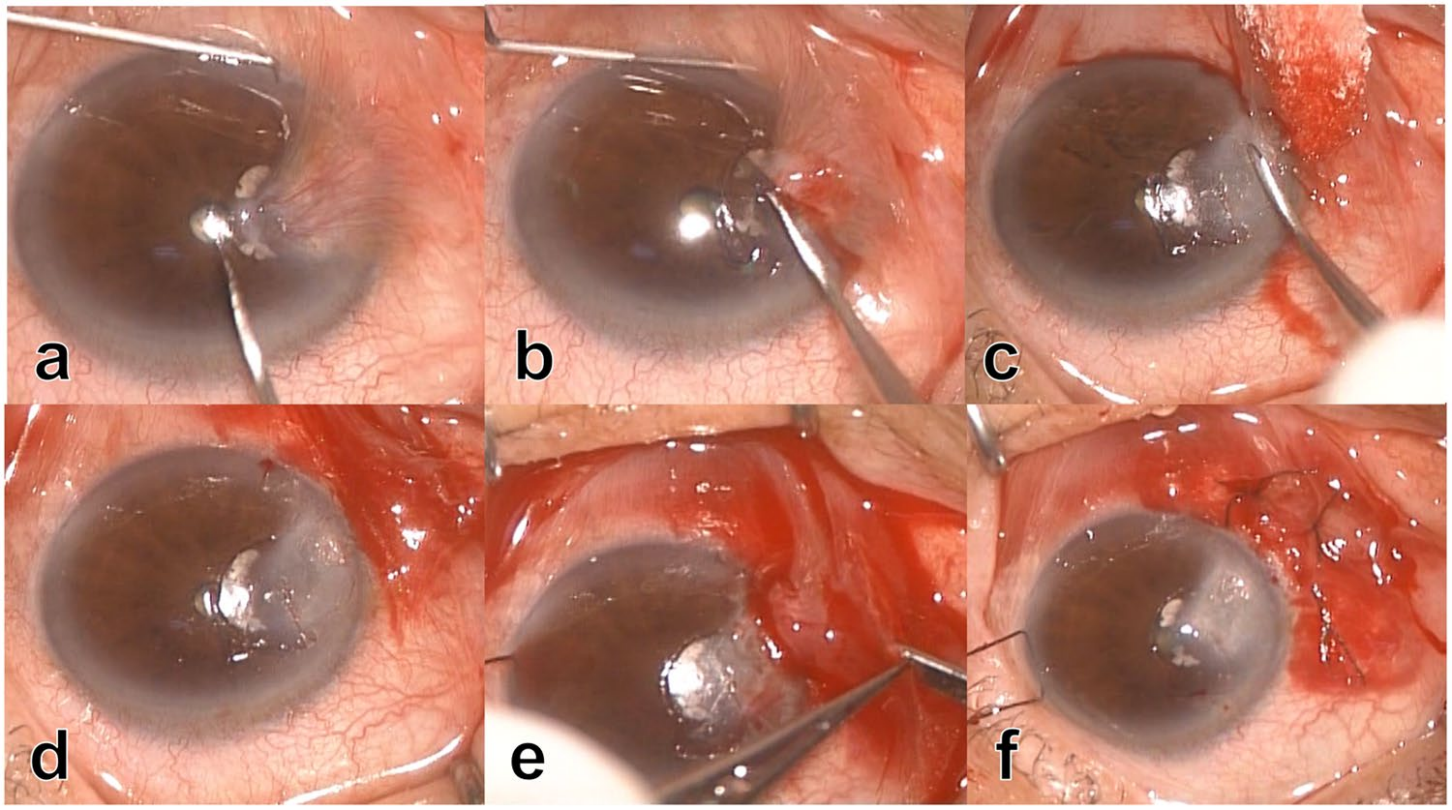
Superficial keratectomy involving normal corneal epithelium was performed using a Beaver blade starting central to the pterygium head (**a**) and working peripherally to the limbal region (**b**), with cares taken not to breach the conjunctiva in order to preserve the baggy structure of the pterygium (**c**). The pterygium was lifted and separated from the underling cornea, and then inverted to the conjunctival region (**d**). A small conjunctival incision was made on the pterygium body approximately 3~4 mm from the limbus, through which a balanced salt solution was irrigated into the subconjunctival space (**e**). The pterygium body was ballooned and unfolded to restore the conjunctival figure, followed by three interrupted 8–0 virgin silk sutures placed to secure the inverted conjunctiva in place (**f**).

After local anaesthesia with 4% lidocaine injected subconjunctivally, superficial keratectomy involving normal corneal epithelium was performed using a Beaver blade starting central to the pterygium head and working peripherally to the limbal region (Fig. 1). At this point, cares should be taken not to breach the conjunctiva in order to preserve the baggy structure of the pterygium from the head to the body. The pterygium was lifted and separated from the underling cornea, but was not trimmed away. The freed pterygium head was then inverted and retracted to the nasal conjunctival region. A small conjunctival incision was made on the pterygium body approximately 3~4 mm from the limbus, through which a balanced salt solution was irrigated into the subconjunctival space. With the irrigation, the pterygium body was ballooned and unfolded to restore the conjunctival figure. Two or three interrupted 8–0 virgin silk sutures were placed to secure the inverted conjunctiva in place. Sutures were removed 8 to 10 days after surgery. After surgery, all patients received topical application of 0.5% levofloxacin hydrate and 0.1% fluorometholone four times a day for 2 months. During and after surgery, no other adjunctive treatments were used, such as mitomycin C, 5-Fluorouracil, or beta therapy (radiotherapy).

Postoperatively, the recurrence of pterygia was judged on the basis of an operation site grading system as previously proposed^7^; grade 1 indicates a normal appearance of the operative site, grade 2 indicates the presence of some fine episcleral vessels extending up to but not beyond the limbus, but without any fibrous tissue, grade 3 indicates the presence of additional fibrous tissues in the area without invading the cornea, and grade 4 represents a true recurrence with a fibrovascular tissue invading the cornea across the limbus. Grades 1 to 3 were considered as “no-recurrence” and grade 4 was considered as “recurrence”^7,8^. The recurrence rate of pterygium was evaluated using the Kaplan-Meier survival analysis involving all 75 eyes. In those patients who were followed up for longer than 6 months after surgery, the status of postoperative nasal conjunctiva was assessed by recording the appearance of vascular loop at the limbus, palisades of Vogt, and pinguecula on slitlamp microscope.

All surgeries were performed by a single surgeon (F.Y.). The study protocol was reviewed and approved by the Institutional Review Board of Yoshitomi Eye Center. This study was conducted in accordance with the Declaration of Helsinki. An informed consent was obtained from all patients in a written form. This study was registered with the University Hospital Medical Information Network Clinical Trials Registry (UMIN-CTR; identification No. UMIN000032001, 30/03/2018).

## Results

**Figure 2:**
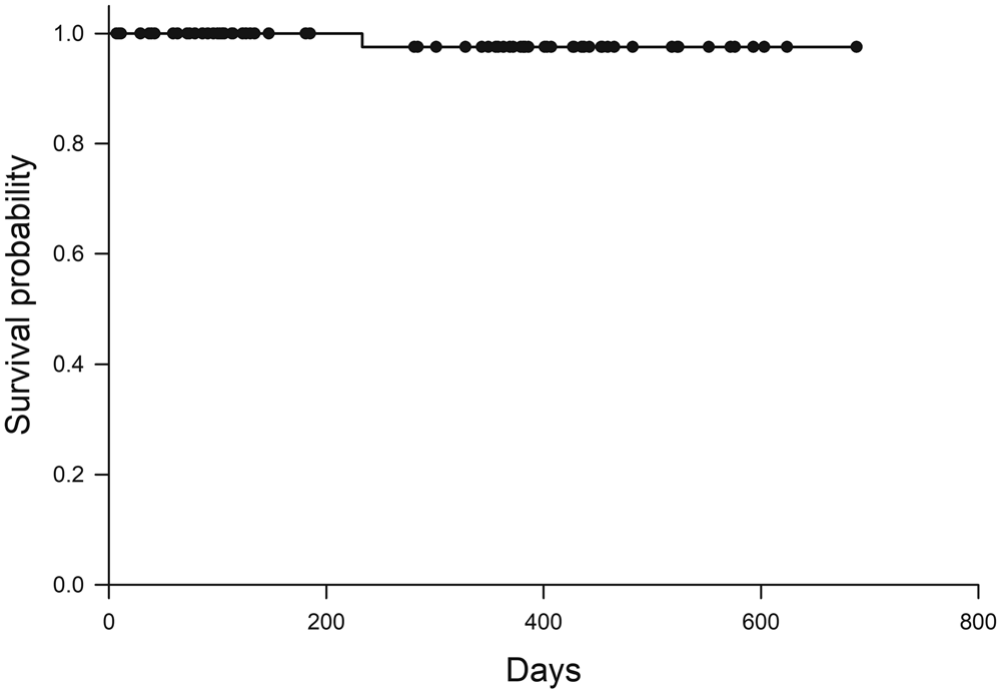
Cumulative probability of non-recurrence after pterygium surgery. Among 75 eyes operated on, one eye developed recurrence at 233 days postoperatively.

There were no intraoperative and postoperative complications related to surgery. Postoperative examination of the surgical site at the final visit revealed that there were 62 cases (82.3%) of grade 1, 11 cases (14.7%) of grade 2, 1 case (1.3%) of grade 3, and 1 case (1.3%) of grade 4. Grades 3 and 4 were observed 382 and 233 days after surgery, respectively. In both eyes, an unintended conjunctival hole was made during the separating process of pterygium from the cornea at the time of surgery. Reoperation was not performed in any eyes. The Kaplan-Meier survival curve of probability of non-recurrence of pterygium is shown in Fig. 2. The recurrence rate at 1 year postoperatively was calculated to be 2.4%.

**Figure 3:**
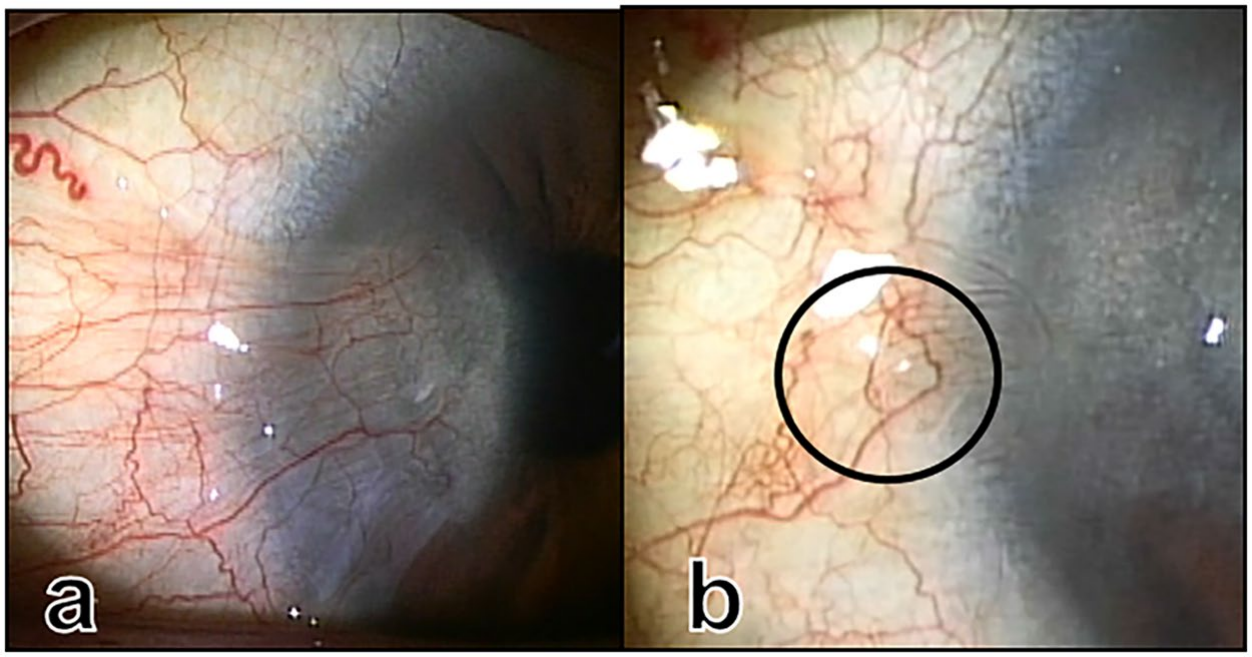
Photographs before (**a**) and 4 months (**b**) after surgery. The vascular loop was seen after surgery (circle).

**Figure 4:**
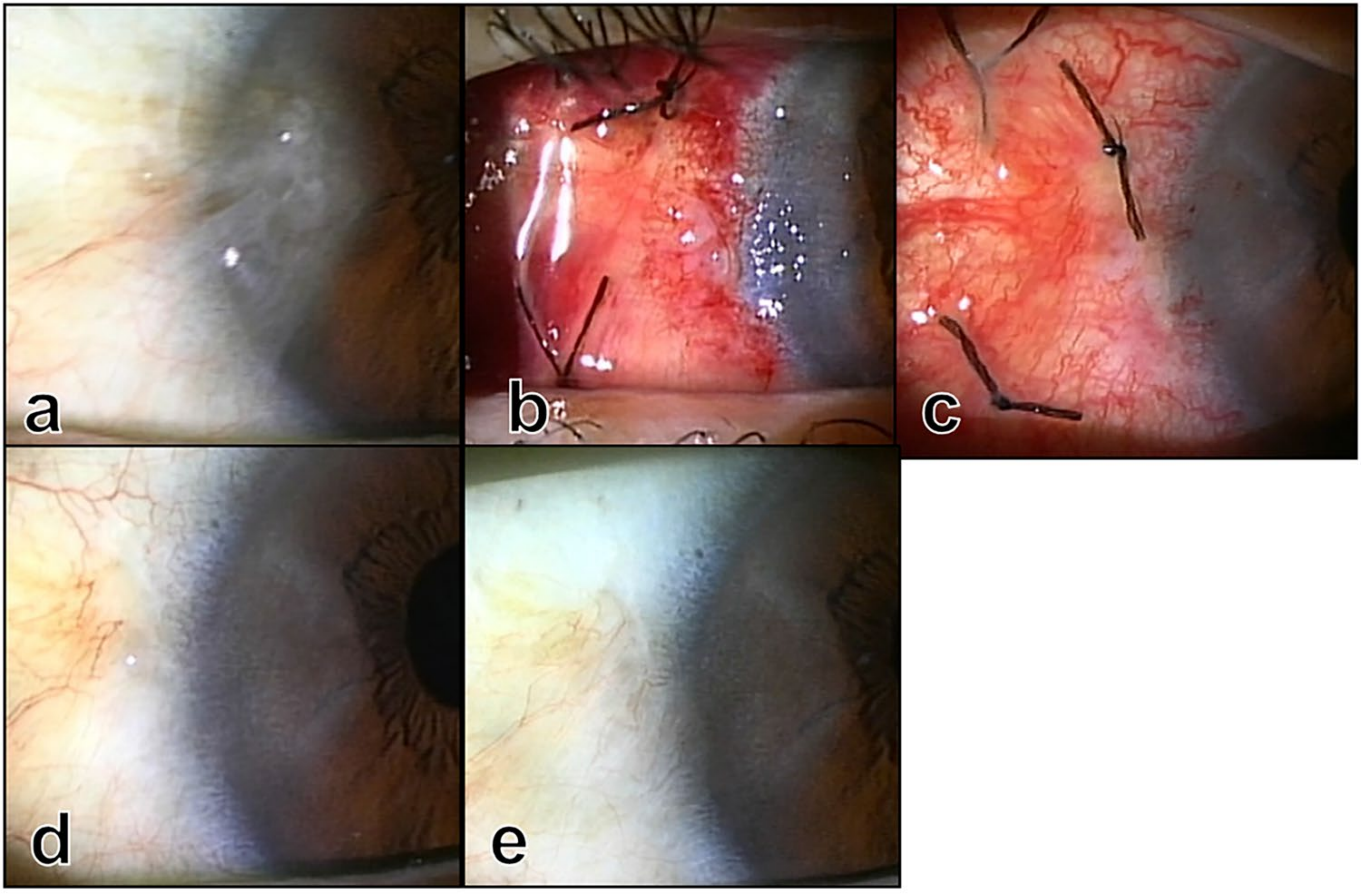
Photographs before (**a**) and immediately (**b**), 8 days (**c**), 1 month (**d**), and 1 year (**e**) after surgery. The palisades of Vogt appeared on the nasal limbus (**d,e**).

**Figure 5:**
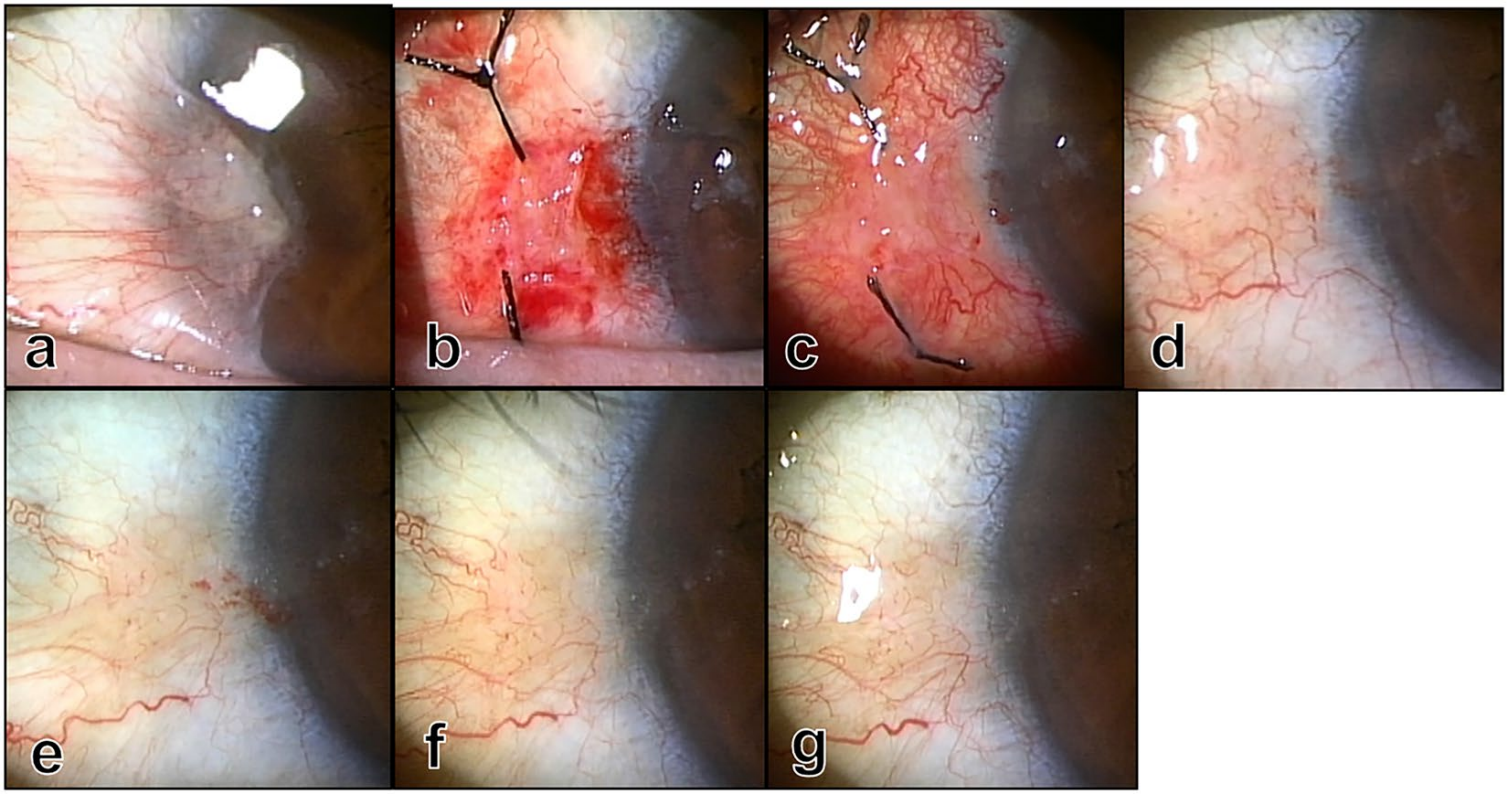
Photographs before (**a**) and immediately (**b**), 8 days (**c**), 1 month (**d**), and 8 months (**e**), 1 year (**f**), and 1 year and 6 months (**g**) after surgery. The pinguecula was found on the nasal conjunctiva (**d,e,f,g**).

In 43 eyes of 43 patients who were followed up for longer than 6 months after surgery, the vascular loop (Fig. 3), which is characteristic of normal limbal structure^9^, appeared on the nasal conjunctiva in 41 eyes (95.3%) after surgery. The palisades of Vogt (Fig. 4) and the pinguecula (Fig. 5) were found on the nasal limbus in 13 eyes (30.2%) and 8 eyes (18.6%), respectively.

## Discussion

We developed the current surgical technique aiming to reduce the rate of recurrence rate of pterygium after surgery. For that end, we tried to restore the physiological structure of the conjunctiva as much as possible by inverting the pterygium head from the cornea to the conjunctival region. This is a new concept which is different from the conventional “cut and remove” strategies. We believed that restoration of conjunctival physiological structure will help maintain conjunctival barrier function and then suppress pterygium recurrence postoperatively. The Kaplan-Meier survival analysis indicated that the pterygium recurrence rate at 1 year postoperatively was 2.4%. This result is favorably compared with those of previous studies; a Cochrane systematic review evaluated 20 randomized controlled trials with 1,866 patients (1,947 eyes) and showed that pterygium recurrence 6 months after surgery ranged from 3.3% to 16.7% in eyes treated with the conjunctival autograft technique and 6.4% to 42.3% in eyes operated on with amniotic membrane transplant^10^.

For the success of this procedure, it is extremely important to avoid making a breach in the conjunctiva during the separation process of pterygium from the cornea. Among the 75 eyes in our study, an unintended conjunctival hole was made in 2 eye (2.7%), which eventually developed to grades 3 and 4 after surgery. In other 73 eyes, there was no break in the pterygium wall and no fibrous tissues developed after surgery. These results indicate that when subconjunctival fibrous tissue is contained within the intact conjunctival epithelium, the pterygium is not likely to recur after the primary surgery. On the other hand, once the conjunctival barrier is disrupted, subconjunctival fibroblasts are exposed and proliferate, leading to recurrence of pterygium.

Our technique is completely different from McReynolds’ operation^11^. McReynolds’ operation consists of dissecting the head with the attached neck and part of the body of the pterygium from its bed. The apex of the pterygium is then transplanted into the lower fornix. This technique had very high rate of recurrence^12^, because the bulbar conjunctival defect at the perilimbal site was left uncovered and the continuous conjunctival barrier was compromised by the surgery. In contrast, our technique does not expose the sclera and retains the continuous conjunctival barrier function.

After surgery, the vascular loop, which is characteristic of normal limbal structure^9^, appeared on the nasal conjunctiva in 95.3% of eyes. The palisades of Vogt and the pinguecula were found postoperatively on the nasal limbus in 30.2% and 18.6% of cases, respectively. These structures are rarely seen after conventional pterygium excision surgery. In addition, the postoperative bulbar conjunctiva appeared almost normal without any scar formation in most of our cases.

Our surgical technique is very easy and simple. There is no need for conjunctival flap, autograft, transplantation of amniotic membrane, and adjunctive therapies, such as mitomycin C, 5-Fluorouracil, or beta therapy (radiotherapy). The conjunctival and subconjunctival tissues are not removed, and hemostasis is not necessary. Only a few anchoring sutures with 8–0 virgin silk are required. The most important precaution is to preserve the conjunctival structure when separating the pterygium from the cornea. The pterygium wall should not be breached so that the pterygium body is inverted onto the conjunctival area as intact as possible. The key to the success of this technique is the performance of superficial keratectomy without compromising pterygium wall during the separation process. Separation can be done between corneal epithelium and stroma, rather than between pterygium and corneal epithelium.

The findings of our study imply some insight into the mechanism of pterygium development. It seems that the pterygium is a baggy structure extending from the conjunctiva onto the cornea. We assume that the first step of pterygium development is detachment of the conjunctiva-Tenon’s capsule-scleral adhesion at the limbus due to aging or ultraviolet radiation. Then, the conjunctival tissue becomes more mobile, which encroaches onto and adheres to the cornea surface following some form of corneal epithelial damages. Our surgical approach aims to reverse this process by blunt dissection of pterygium from the cornea and inversion of the pterygium head to the conjunctival site. When successful, physiological features of the conjunctiva are restored, such as the vascular loop and the palisades of Vogt.

The present study has several limitations. First, the study population size was small and postoperative observation period may not have been long enough. We operated on 75 eyes, but only 43 eyes were followed up for 6 months or longer after surgery. Since pterygium is a benign disease, many patients do not return to postoperative check-up after successful surgery. Second, there was no control group. Since we abandoned excision and removal techniques to treat pterygium several years ago, it was not practically possible to conduct a randomized control trial to compare the current and other surgical techniques. Third, our surgical technique is obviously not effective to treat recurrent pterygium after excision surgery, in which the initial pterygium removal surgery has damaged conjunctival barrier function and baggy structure of the pterygium is not maintained. More aggressive treatment strategies are needed to treat recurrent pterygia. On the other hand, the current technique was effective for primary pterygia regardless of its size and grading.

In conclusion, we developed a new surgical approach to treat pterygium; pterygium head inversion technique. By separating the pterygium from the cornea and inverting the intact pterygium head onto the nasal conjunctival site, the conjunctiva restored near physiological status after surgery. The recurrence rate was very low.

## Data Availability

The datasets generated and analysed during the current study were uploaded to Springer Nature Research Data Support. Yoshitomi, Fumiaki; Oshika, Tetsuro; Postoperative examination results from 75 patients undergoing a novel procedure to correct pterygium (2018). figshare. 10.6084/m9.figshare.6282668.
